# Supplementary material for: Content-rich biological network constructed by mining PubMed abstracts
Source: BMC Bioinformatics. 2004 Oct 8;5:147. doi: 10.1186/1471-2105-5-147 (PMC528731; doi:10.1186/1471-2105-5-147)
Supplement: Additional File 5 — The original Chilibot query results of the term "long-term potentiation (LTP)" and 22 other terms, limiting the latest references analyzed to the years 1990, 1995, 2000, and 2004. [file 1471-2105-5-147-S5.bz2 › chilibotAdditionalFile5/ltp2000/html/CREB_NMDA.html]

 


 **CREB** and **NMDA** 
  
Found 32 abstracts in PubMed,  **30 abstracts were retrieved and analyzed**.  


---

 Search Google  |
 PDF files only 
|  EDU domain only 

---

**Interactive relationship** (e.g. stimulation, inhibition, etc)

- At all stages of development  **NMDA**  stimulation induced a rapid phosphorylation of  **CREB**  on Ser 133 phospho  **CREB** .  Ref: 10804193 J Neurosci, 2000
- At 7 d in vitro 7 DIV phospho  **CREB**  remained elevated 2 hr after strong  **NMDA**  stimulation.  Ref: 10804193 J Neurosci, 2000
- **NMDA**  and D1 receptors regulate the phosphorylation of  **CREB**  and the induction of c fos in striatal neurons in primary culture.  Ref: 9068120 Synapse, 1997
- Elevation of calcium influx by stimulation of L type calcium channels,  **NMDA** , or KCl induced strong  **CREB**  phosphorylation in the dorsal striatum but not in the olfactory tubercle.  Ref: 9539803 Proc Natl Acad Sci U S A, 1998
- In primary cultures of striatal neurons, glutamatergic activation of ERK and one of its transcription factor targets,  **CREB** , showed a calcium dependence typical of  **NMDA**  receptor mediated responses.  Ref: 10349832 J Neurochem, 1999
- These results support the proposal that an intracerebroventricular injection of  **NMDA**  may selectively potentiate DNA binding activities of both AP1 and  **CREB**  through activation of the  **NMDA**  receptor complex in mouse brain.  Ref: 8035176 J Neurochem, 1994
- Here, we show that activation of  **NMDA**  receptors and voltage sensitive calcium channels leads to  **CREB**  mediated transcription in cortical neurons via a mechanism regulated by  **CREB**  binding protein CBP.  Ref: 10230799 Neuron, 1999
- Moreover, at 14 DIV...  **NMDA**  receptor stimulation induced a dephosphorylation of  **CREB**  that previously had been phosphorylated by KCl depolarization or forskolin, suggesting an  **NMDA**  receptor dependent activation of a  **CREB**  phosphatase.  Ref: 10804193 J Neurosci, 2000
- We suggest that, during neuronal maturation,  **NMDA**  receptor activation becomes linked specifically to protein phosphatases that act on Ser 133 of  **CREB** .  Ref: 10804193 J Neurosci, 2000
- Moreover, blockade of  **NMDA**  receptors diminishes the ability of dopamine to induce phosphorylation of the cyclic AMP responsive element binding protein  **CREB** .  Ref: 8753884 J Neurosci, 1996
- Glutamatergic induction of  **CREB**  phosphorylation and Fos expression in primary cultures of the suprachiasmatic hypothalamus in vitro is mediated by co ordinate activity of  **NMDA**  and non  **NMDA**  receptors.  Ref: 9918228 J Neuroendocrinol, 1999
- Treatment of the tissue with a mixture of glutamatergic agonists N methyl D aspartate  **NMDA** , amino methyl proprionic acid AMPA and kainate, all at 1 microM, or native glutamate 1 microM had no effect on the total  **CREB**  signal, but increased the pCREB signal, indicative of agonist stimulated phosphorylation of  **CREB**  on Ser133.  Ref: 9753174 Eur J Neurosci, 1998
- L Glu,  **NMDA** , and KA evoked a dose and time dependent increase in AP 1 DNA binding activity and had no effect on  **CREB**  binding.  Ref: 7650753 J Neurosci Res, 1995
- Calcium influx through postsynaptic  **NMDA**  receptors can stimulate neuronal gene expression via signaling pathways such as the Ras MAP kinase MAPK pathway and the transcription factor cAMP response element binding protein  **CREB** .  Ref: 10804193 J Neurosci, 2000
- There was no developmental change in the time course of phospho  **CREB**  induction that followed KCl depolarization or PKA activation, nor was there a developmental change in the time course of phospho Erk1 2 induced by  **NMDA**  receptor activation.  Ref: 10804193 J Neurosci, 2000
- Direct application of  **NMDA**  to striatal cells in culture caused a rapid increase in the expression of c fos as well as an increase in the phosphorylation of the transcription factor CRE binding protein  **CREB** .  Ref: 9068120 Synapse, 1997
- The mode of calcium entry L type calcium channels versus  **NMDA**  receptors differentially controls the CBP recruitment step to  **CREB** , providing a molecular basis for the observed calcium channel type dependent differences in gene expression.  Ref: 10230798 Neuron, 1999
- In contrast,  **NMDA**  virtually did not alter DNA binding activities of both  **CREB**  and Myc in discrete structures of murine brain under similar experimental conditions.  Ref: 7833794 Neurochem Int, 1994
- Both formalin evoked  **CREB**  phosphorylation and c Fos expression in the spinal cord were suppressed by pretreatment with the  **NMDA**  receptor antagonist MK 801 3.5 mg kg, i.p. or halothane anesthesia.  Ref: 9030636 J Neurosci, 1997

**Parallel relationship** (e.g. studied together, co-existance, homology, etc.)

- The effect of L glutamate L Glu and its structural analogs N methyl D aspartate  **NMDA** , quisqualate QA, and kainate KA on the DNA binding activity of the Activator Protein 1 AP 1 and the calcium cAMP Responsive Element Binding Protein  **CREB**  families of transcription factors was examined in cultured chick retinal Müller glia cells.  Ref: 7650753 J Neurosci Res, 1995
- The effects of  **NMDA**  and nitric oxide synthase NOS antagonists on Glu mediated induction of P  **CREB**  lir paralleled their inhibition of phase shifting.  Ref: 8987789 J Neurosci, 1997
- Here, we provide evidence for the participation of hippocampal PKA  **CREB**  and MAPK Elk 1 pathways, via activation of  **NMDA**  receptors, in memory formation of a one trial avoidance learning in rats.  Ref: 10719213 Brain Res Mol Brain Res, 2000
- An injection of saline transiently increased binding of both probes for activator protein 1 AP1 and cyclic AMP response element binding protein  **CREB**  30 min after the injection, and  **NMDA**  was effective in inducing a more potent increment of binding of both probes 1 5 h after the injection than did saline.  Ref: 8035176 J Neurochem, 1994
- We analyzed MAPK Erk1 2 and  **CREB**  activation in response to  **NMDA**  receptor stimulation during the development of hippocampal neurons in culture.  Ref: 10804193 J Neurosci, 2000
- The work presented here attempts to identify in striatal neurons the pathways that mediate cAMP response element binding protein  **CREB**  phosphorylation and gene expression in response to  **NMDA**  receptor activation.  Ref: 10414964 J Neurosci, 1999
- Pretreatment with the  **NMDA**  receptor antagonist 5 methyl 1 1 dihydro 5H dibenzo a, d cyclohepten 0 imine maleate completely prevented activation of  **CREB** .  Ref: 10377374 J Neurosci, 1999
- The induction of c fos and  **CREB**  phosphorylation following  **NMDA**  were unaffected by inhibition of protein kinase C.  Ref: 9068120 Synapse, 1997
- Together, these data couple nocturnal light, Glu,  **NMDA**  receptor activation and NO signaling to  **CREB**  phosphorylation in the transduction of brief environmental light stimulation of the retina into molecular changes in the SCN resulting in phase resetting of the biological clock.  Ref: 8987789 J Neurosci, 1997
- These observations are consistent with the hypothesis that calcium calmodulin dependent phosphorylation of  **CREB**  induced by  **NMDA** , or cAMP dependent phosphorylation of  **CREB**  induced by D1 agonists, underlie the induction of c fos seen following activation of these receptors in striatal neurons.  Ref: 9068120 Synapse, 1997
- Developmentally regulated  **NMDA**  receptor dependent dephosphorylation of cAMP response element binding protein  **CREB**  in hippocampal neurons.  Ref: 10804193 J Neurosci, 2000
- We found that neither AMPA kainate receptors nor  **NMDA**  receptors were able to stimulate independently a second messenger pathway that led to  **CREB**  phosphorylation or c fos gene expression.  Ref: 10414964 J Neurosci, 1999
- Learning associated activation of nuclear MAPK,  **CREB**  and Elk 1, along with Fos production, in the rat hippocampus after a one trial avoidance learning abolition by  **NMDA**  receptor blockade.  Ref: 10719213 Brain Res Mol Brain Res, 2000
